# Altered Serum Metabolic Profile Assessed by Advanced 1H-NMR in Breast Cancer Patients

**DOI:** 10.3390/cancers13174281

**Published:** 2021-08-25

**Authors:** Josep Gumà, Jose Adriá-Cebrián, Belen Ruiz-Aguado, Cinta Albacar, Josefa Girona, Ricardo Rodríguez-Calvo, Neus Martínez-Micaelo, Eric W. F. Lam, Luis Masana, Sandra Guaita-Esteruelas

**Affiliations:** 1 Department of Oncology, Hospital Universitari de Sant Joan, 43204 Reus, Spain; jguma@grupsagessa.cat (J.G.); joseadriacebrian@gmail.com (J.A.-C.); belen.ruiz@grupsagessa.com (B.R.-A.); cintarosa.albacar@grupsagessa.com (C.A.); 2 Center for R&D&I in Nutrition and Health, Institut de Investigació Sanitaria Pere Virgili (IISPV), Avda. de la Universitat, 1—Second Floor, 43204 Reus, Spain; josefa.girona@urv.cat (J.G.); ricardo.rodriguez@urv.cat (R.R.-C.); neus.martinez@urv.cat (N.M.-M.); luis.masana@urv.cat (L.M.); 3 Research Unit on Lipids and Atherosclerosis, Institut de Investigació Sanitaria Pere Virgili (IISPV), Universitat Rovira i Virgili, 43204 Reus, Spain; 4 Spanish Biomedical Research Centre in Diabetes and Associated Metabolic Disorders (CIBERDEM), Institute of Health Carlos III, 28029 Madrid, Spain; 5 State Key Laboratory of Oncology in South China, Collaborative Innovation Center of Cancer Medicine, Sun Yat-sen University Cancer Center, Guangzhou 510060, China; ericlam7314@gmail.com

**Keywords:** lipoproteins, breast cancer, triglycerides

## Abstract

**Simple Summary:**

Previously, our group demonstrated high FABP4 circulating levels in breast cancer (BC) patients. Moreover, increased cholesterol and triglycerides (TG) were found. To deeply analyse the lipid metabolism in our BC cohort, lipid and low molecular weight metabolomics processes are performed in 240 women (171 BC and 69 control women). This paper provides original data related to a novel link between TG-enriched particles and BC. The main result of this study is that TG-enriched particles and some branched amino acids, as well as tyrosine and alanine, are positively associated with BC. This suggests that BC patients have a different metabolic signature that could be used for better stratification and treatment. To our knowledge, this is the first time that advanced NMR profiling has been used to identify relevant and specifically altered lipid and amino acid metabolites in BC serum samples, which could be used for early and reliable diagnosis and prognosis.

**Abstract:**

Background: Altered lipid metabolism has been described in some types of cancer. To analyse in depth the metabolic modifications in breast cancer patients, advanced 1H-nuclear magnetic resonance was performed in these patients. The main objective of this paper was to define a specific lipidomic signature for these cancer patients. Materials and methods: Serum from 240 women (171 breast cancer patients and 69 control women) were studied and analysed by nuclear magnetic resonance. Results: Triglyceride-enriched particles, specifically very low-density lipoprotein triglycerides, intermediate-density lipoprotein triglycerides, low-density lipoprotein triglycerides, and high-density lipoprotein triglycerides, were positively associated with breast cancer. Moreover, alanine, tyrosine, and branched amino acids were also associated with increased risk of breast cancer. Conclusions: Breast cancer patients showed a modified metabolome, giving a very interesting tool to draw different radar charts between control women and breast cancer patients. To our knowledge, this is the first time that advanced nuclear magnetic resonance profiling has been used to identify relevant and specifically altered lipid or amino acid metabolites in BC serum samples. The altered metabolic signature could be analysed for early and reliable BC patient diagnosis and prognosis.

## 1. Introduction

Breast cancer is the most frequent tumour in women and the second leading cause of cancer-related deaths [1]. Recently, energy metabolism reprogramming has been described as an emerging hallmark in cancer [2,3]. Glucose and glutamine metabolism processes were identified as important metabolic changes in cancer cells [4]; however, recently alterations in the metabolism and regulation of lipids have gained increasing interest because of the definition of new roles for lipids in cancer progression [5]. These lipid modifications include lipid uptake, storage, lipogenesis, and lipolysis [6,7]; actually, cancer cells increase exogenous lipid uptake and endogenous synthesis in order to satisfy their needs for these molecules [3].

Lipids can promote cancer progression at the cellular level, although the epidemiological association is not clear. In fact, cholesterol has a crucial role in cell membrane regulation in mammalian cells, through modulating signal transduction [8]. Moreover, intracellular cholesterol levels are controlled by new biosynthesis, as well as extracellular and intracellular transport. Cholesterol levels are critical in some classical metabolic diseases, such as atherosclerosis, although can also be important for the pathogenesis of others, including cancer [9]. Moreover, cholesterol is a precursor for oestrogens and androgens, both of which are involved in the processes of tumour initiation and progression. Furthermore, oxysterols, molecules derived from cholesterol, are able to increase cancer cell growth and metastasis [8]. Indeed, lipid rafts are essential for cancer signalling and are enriched in cholesterol. Some modifications in their composition can lead to changes in signal transduction and cancer progression. For these reasons, cholesterol has increasing importance in the cancer development process [10].

In spite of the growing availability of these molecular data, there are still epidemiological discrepancies regarding lipid levels and cancer [10,11]. Particularly, opposing results have been found related to the association of lipids and breast cancer [12,13]. In concordance, despite the fact that there have been promising results regarding the use of statins for cancer treatment, the results are quite variable depending on the cancer type [8].

Metabolomic profiling has appeared as a good identification and quantification method for metabolic products such as diagnostic and prognostic biomarkers for certain disorders and diseases [14]. Traditional methods for lipid measurement only quantify circulating lipids by lipoparticle concentration, although their size, density, and triglyceride (TG) composition are usually not analysed during diagnostic analysis. Similarly, conventional measurements of circulating lipid particles only analyse the amounts of cholesterol in the lipid particles, although these methods are not focused on their composition (i.e., TG and phospholipids), particles size, or subclass concentration. Equally, circulating TG are usually measured in terms of total TG concentration and not based on their lipoparticle subclass [15]. Nevertheless, the relationship between lipids and cancer is affected by many factors other than lipid concentration alone; thus, the main goal of this study was to analyse lipoprotein particle subclasses, size, and composition characteristics in breast cancer patients in order to identify potential biomarkers for breast cancer diagnosis and prognosis.

## 2. Materials and Methods

### 2.1. Studied Population

In total, 240 individuals were enrolled into this metabolomics study, which included standard clinical biochemistry analysis. There were 171 breast cancer patients and 69 control subjects. Breast cancer patient samples were collected by the department of oncology from Hospital Universitari Sant Joan de Reus after breast cancer diagnosis. Common control subjects were selected from the same family to match age, body mass index (BMI), and geographical area characteristics where possible. Regular examinations were performed and recorded. The project was approved by the Hospital Ethical Committee (reference number: 99-05-20/04-5), and the subjects signed their written consent to participate in the study and accepted the publication of the results.

BMI was calculated as the body weight (kg) divided by the body height squared (m^2^).

### 2.2. Blood Sample Collection and Storage

The blood samples were obtained after overnight fasting. Serum aliquots were stored at −80 °C in the Biobank of our centre in the Institut d’Investigació Sanitària Pere Virgili (IISPV) until their use.

### 2.3. Biochemical Analysis

Standard biochemical parameters were analysed previously [16]. Cluster of differentiation 36 (CD36) was analysed using a commercial ELISA kit (R&D Systems, Vitro, Madrid, Spain). CETP protein and activity were evaluated using commercial ELISA kits (Cusabio, Deltaclon, Madrid, Spain and Sigma-Aldrich, Merck, Madrid, Spain respectively).

### 2.4. Lipoprotein Analysis by NMR Spectroscopy (Advanced Lipoprotein Profile)

The serum samples were sent to Biosfer Teslab in dry ice for the NMR analysis. Here, 200 μL was transferred into NMR tubes with phosphate buffer. High-resolution 1H-NMR spectroscopy data were acquired on a Bruker 600 MHz spectrometer, while 1D nuclear Overhauser effect spectroscopy (NOESY, 4 scans) and Carr–Purcell–Meiboom–Gill (CPMG, 64 scans) analysis were used to characterise small molecules such as amino acids and sugars. LED diffusion (Diff) experiments (32 scans) were used to detect larger molecules such as lipoproteins and glycoproteins compounds. All of the sequences were run at 37  °C. The lipid concentrations, sizes, and particle numbers of the four main classes of lipoproteins and the particle numbers of nine subclasses were analysed as previously reported [17]. Briefly, particle concentrations and diffusion coefficients were obtained using the amplitudes and attenuation of their methyl group NMR signals using the 2D diffusion-ordered 1H NMR spectroscopy (DSTE) pulse. The methyl signal was surface-fitted with 9 lorentzian functions associated with each lipoprotein subclasses. The area was related to the lipid concentration of each lipoprotein and the size was calculated from their diffusion coefficient.

The coefficient between the lipid volume and the particle volume of a given class provided the subclass particle concentration. The common conversion factors used to transform concentration units into volume units gave the lipid volumes [18]. Finally, weighted average particle sizes were calculated by summing the known diameter of each subclass multiplied by its relative percentage of the subclass particle number.

### 2.5. Low Molecular Weight Metabolites Analysis

The CPMG spectra were phased, baseline-corrected, and referenced before performing the automatic metabolite profiling as previously reported using Dolphin software. The 14 low molecular weight metabolites (LMWMs) were identified and quantified. Identifications were analysed for all resonances along the spectra and quantification was performed using line–shape fitting methods on one of the signals.

### 2.6. Lipid Extraction

Lipophilic extracts were obtained from two 100 μL aliquots of freshly thawed plasma using the BUME method with slight modifications. BUME was optimised for batch extractions with diisopropyl ether (DIPE). This procedure was performed with a BRAVO liquid handling robot, involving drying of the upper lipophilic phase in a Speedvac until evaporation of organic solvents occurred and freezing at −80 °C for further NMR analysis. Lipid extracts were reconstituted in a solution of CDCl3–CD3OD–D2O (16:7:1, *v*/*v*/*v*) containing tetramethylsilane (TMS) at 1.18 mM and transferred into 5 mm NMR glass tubes. An Avance III-600 Bruker spectrometer was used to measure the 1H-NMR spectra at 600.20 MHz. A 90° pulse with a water presaturation sequence (zgpr) was used. Quantification of lipid signals was carried out with LipSpin6, an in-house software based on Matlab. Resonance assignments were performed based on literature values [19].

### 2.7. Statistical Analysis

The results are expressed as the means ± standard deviation (SD) for normally distributed data, the medians (interquartile range) for data that were not normally distributed, and frequencies for categorical data. The differences between groups were assessed using Student’s t test, the Mann–Whitney U test, or chi-square tests. Binary logistic regression analysis was used to calculate the odds ratios (ORs) in serum parameters associated with the presence of breast cancer. In order to facilitate comparisons, the traits were standardised (metabolic marker divided by its standard deviation) (Holmes et al. 2018). Finally, we depicted the adjusted ORs and 95% confidence intervals (CIs) for each 1-SD higher metabolic measure.

SPSS software was used to perform the statistical analyses (IBM SPSS Statistics, version 20.0, North Castle, New York, http://www.ibm.com). Here, *p* values <0.05 were considered to be statistically significant.

## 3. Results

### 3.1. Initial Characteristics of the Study Population

Among the 240 participants with biochemical measurements, the mean age of breast cancer patients 44 (37–50) was similar to control women 43 (38–54) (*p* = n.s). The same trends were found regarding ages of menarche for control women (12; 11–13) and breast cancer patients (12; 12–14) (*p* = n.s), numbers of children for control women (2; 1–2) and breast cancer patients (2; 1–2) (*p* = n.s), and body mass index values for breast cancer patients (24.02; 22.20–28.13) and control patients (25.07; 22.72–28.36) (*p* = n.s). Finally, no significant differences were found in the percentages of menopausal women, with 30.3% in control women versus 20.2% in breast cancer patients (*p* = n.s)

For lipids measured using clinical chemistry, breast cancer patients had higher mean concentrations of total cholesterol (208.06 ± 31.40 in breast cancer patients compared to control women 194.18 ± 28.31; *p* = 0.002), Apo B100 101 (85–118 in breast cancer patients compared to 91 (78–104) in control women; *p* = 0.008), and triglycerides (86.73 (67.69–120.16) in breast cancer patients compared to 76.55 in control women (56.83–98.74); *p* = 0.004). In contrast, Apo A1, CETP, and CD36 protein concentrations were broadly similar between breast cancer and control women. Moreover, no significant differences were found in CETP activity (Table 1).

### 3.2. Serum Metabolome Changes

**Figure 1:**
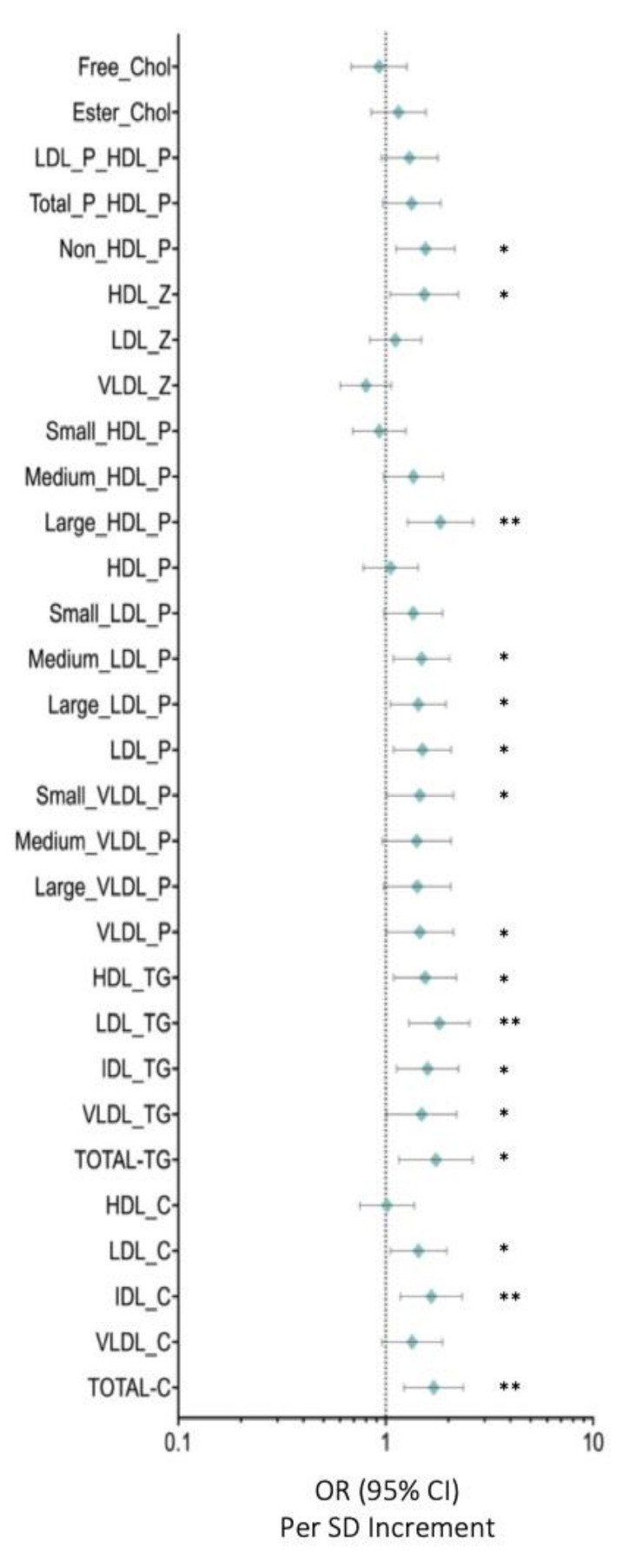
Serum metabolome. Data are presented as OR (95% CI) per 1-SD higher metabolic marker. The plot shows the OR of the effect of a 1-SD-deviation increase and the errors bars represent 95% of the effect estimate. The vertical line marks an OR of 1, i.e., no effect of the exposure on BC risk. Models are adjusted for body mass index and age. Abbreviations: Total-C, total cholesterol; VLDL-c, very low-density lipoprotein cholesterol; IDL-c, intermediate-density lipoprotein cholesterol; LDL-c, low-density lipoprotein cholesterol; HDL-c, high-density lipoprotein cholesterol; Total-TG, total triglycerides; VLDL-TG, very low-density lipoprotein triglycerides; IDL-TG, intermediate-density lipoprotein triglycerides; LDL-TG, low-density lipoprotein triglycerides; HDL-TG, high-density lipoprotein triglycerides; VLDL-P, very low-density lipoprotein particle; large VLDL-P, large very low-density lipoprotein particle; medium VLDL-P, medium very low-density lipoprotein particle; small VLDL-P, small very low-density lipoprotein particle; LDL-P, low-density lipoprotein particle; large LDL-P, large low-density lipoprotein particle; medium LDL-P, medium low-density lipoprotein particle; Small LDL-P, Small low-density lipoprotein-particle; HDL-P, high-density lipoprotein-particle; Large HDL-P, large high-density lipoprotein particle; medium HDL-P, medium high-density lipoprotein particle; small HDL-P, small high-density lipoprotein particle; VLDL-z, very low-density lipoprotein size; LDL-z, low-density lipoprotein size, HDL-z, high-density lipoprotein size; non-HDL-p, non-high-density lipoprotein particle; T-P_HDL-P, total particles/high density lipoprotein particle; LDL-P_HDL-P, low-density lipoprotein particle/high-density lipoprotein particle; Esterified Chol, esterified cholesterol; Free chol, free cholesterol. Note: *p* values <0.05 were considered to be statistically significant (*p* <0.05 * and *p* < 0.005 **).

Figure 1 depicts the OR for lipid particles and selected covariates in breast cancer compared with those in control women. Total cholesterol (OR: 1.702 (95% CI: 2.365–1.225)) (*p* < 0.005), IDL-C (OR: 1.659 (95% CI: 2.337–1.178)) (*p* < 0.005), LDL-C (OR: 1.440 (95% CI:1.965–1.055)) (*p* < 0.05), total-TG (OR: 1.745 (95% CI: 1.159–2.625)) (*p* < 0.05), VLDL-TG (OR: 2.196 (95% CI: 2.196–1.009)) (*p* < 0.05), IDL-TG (OR: 1.592 (95% CI: 1.131–2.243)) (*p* < 0.05), LDL-TG (OR: 1.812 (95% CI: 2.537–1.294)) (*p* < 0.005), HDL-TG (OR: 1.549 (95% CI: 2.192–1.095)) (*p* < 0.05), VLDL particles (OR: 1.460 (95% CI: 2.121–1.004)) (*p* < 0.05), small VLDL-*p* (OR: 1.462 (95% CI: 2.121–1.008)) (*p* < 0.05), LDL particles (OR: 1.503 (95% CI: 2.068–1.093))(*p* < 0.05), large LDL-*p* (OR: 1.436 (95% CI: 1.951–1.057)) (*p* < 0.05), medium LDL-*p* (OR: 1.489 (95% CI:2.033–1.091)) (*p* < 0.05), large HDL-*p* (OR: 1.834 (95% CI: 2.641–1.274 )) (*p* < 0.005), HDL-Z (OR: 1.533 (95% CI: 2.235–1.051)) (*p* < 0.05), and non-HDL-P (OR: 1.554 (95% CI: 2.152–1.122)) (*p* < 0.05) were associated with higher risks of breast cancer after adjusting for body mass index and age.

### 3.3. HDL Particles Are Altered and Transport a Higher Content of TG in BC Patients

**Figure 2:**
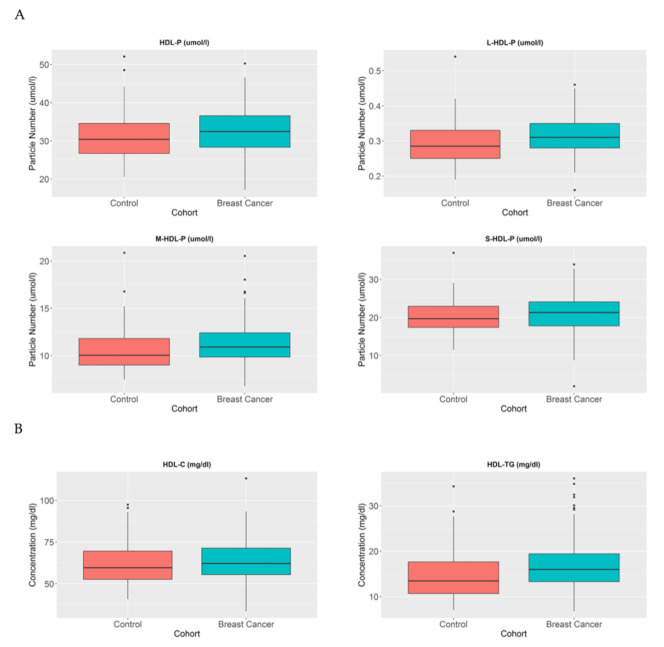
HDL-P, HDL-C, and HDL-TG serum concentrations in BC patients and control women. The blue blots represent BC patients and the red blots represent heathy women. The blots depict the mean and standard error of the mean: (**A**) HDL particles, large HDL particles, medium HDL particles, and small HDL particles; (**B**) HDL-C and HDL-TG. Note: *p* values < 0.05 were considered to be statistically significant. Abbreviations: BC, breast cancer; HDL, high-density lipoprotein.

HDL particles (HDL-P) have been studied in the reverse cholesterol transport context for many years, and they play an important role in the elimination of cholesterol in the body. Different metabolic alterations cause the disruption of HDL profiles in patients [20]. In this study, the number and composition of these particles were analysed in BC patients in order to understand their metabolic effects. HDL particle number profiles were compared between the healthy population and BC women and we observed that despite the fact that there were no differences in HDL-P in breast cancer patients compared to control women (*p* = 0.159), the large HDL-P (*p* < 0.005) and medium HDL-P (*p* < 0.05) were increased in breast cancer patients compare to control women (Figure 2A).

When we analysed these particles in more detail, we observed that their lipid profiles were also altered. In the BC population, the HDL particles transported a higher proportion of triglycerides in comparison with control women (*p* < 0.005) (Figure 2B).

### 3.4. LDL-P and Their Lipid Contents Are Increased in BC Patients

**Figure 3:**
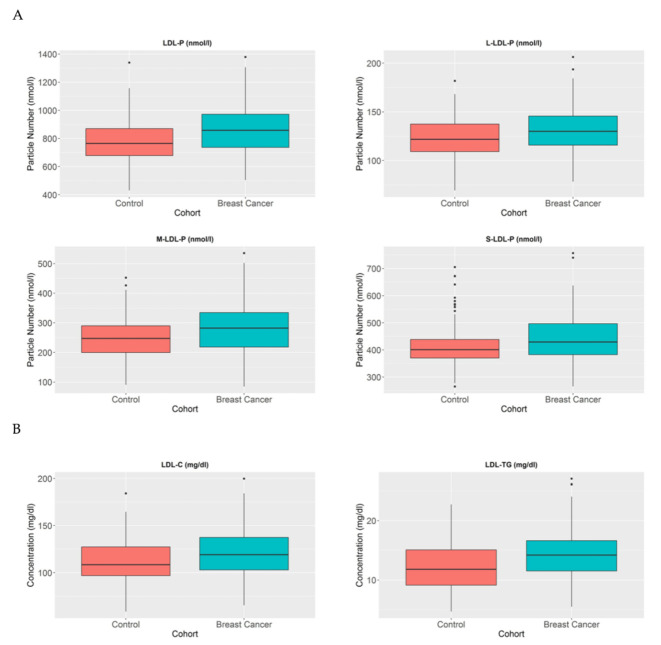
LDL-P, LDL-C, and LDL-TG serum concentrations in BC patients and control women. The blue blots represent BC patients, while the red blots represent heathy women. The blots symbolise the mean and standard error of the mean: (**A**) LDL particles, large LDL particles, medium LDL particles, and small LDL particles; (**B**) LDL-C and LDL-TG. Note: *p* values < 0.05 were considered to be statistically significant. Abbreviations: BC, breast cancer; LDL, low-density lipoprotein.

LDL particles have been established as particles that transport cholesterol from the liver to other parts of the body. Several studies have demonstrated a correlation between higher number of LDL particles and different metabolic diseases, particularly as a crucial factor in atherosclerosis [21]. In the cancer context, some studies have established LDL as a changeable factor, which is increased in patients with BC [10,22]. In agreement, we also detected that BC patients have a higher number of LDL particles compared to healthy individuals (*p* < 0.005) (Figure 3A). Moreover, when we analysed these particles in further detail, we also observed that the large (*p* < 0.005), medium (*p* < 0.005), and small LDL (*p* < 0.05) particles are increased in BC patients compared to control women. When we further analysed the LDL composition in our cohort, we again detected that the LDL particles in BC patients contain a higher amount of cholesterol compared to control women (*p* < 0.005). Moreover, when we studied the TG composition of LDL particles, we also observed a higher TG proportion in LDL particles in BC patients compared to control women (*p* < 0.001) (Figure 3B).

To deeply analyse the lipidomics of breast cancer patients, lipid extraction and analysis by 1H-NMR were performed. No differences were found in lipidomics when breast cancer patient samples were analysed compared to control women.

### 3.5. Low Molecular Weight Metabolites Reveal an Imbalance in Branched Chain Amino Acids As Well As in Tyrosine and Alanine in BC Samples

The demand for glucose is often elevated in cancer patients. In advanced stages of cancer, an increase in proteolysis has been described to meet cancer cells’ augmented energy demands. Moreover, in early stages, alterations in amino acid profiles have also been observed in some cancer types (Lieu et al. 2020).

**Figure 4:**
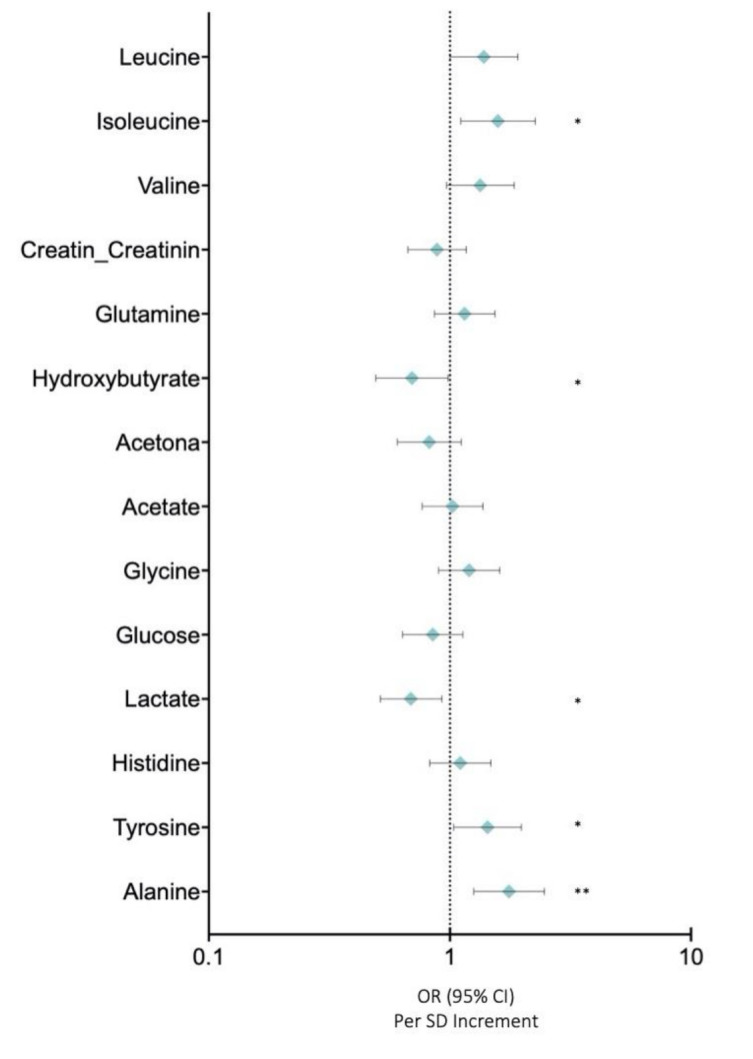
Low molecular weight metabolites. Data are presented as OR (95% CI) per 1-SD higher metabolic marker. Models are adjusted for body mass index and age. Note: *p* values <0.05 were considered to be statistically significant (*p* <0.05 * and *p* < 0.005 **).

In this study, we analysed the profiles of low molecular weight metabolites (LMWMs) in BC patients using NMR in order to investigate the metabolic alterations in our cohort of breast cancer patients. Figure 4 depicts the OR for LMWMs and selected covariates in BC compared with those in control women. Correlation analysis showed that alanine (OR: 1.759 (95% CI: 2.466–1.255)) (*p* < 0.005), tyrosine (OR: 1.432 (95% CI: 1.979–1.036)) (*p* < 0.05), and isoleucine (OR: 1.382 (95% CI: 1.911–1.000)) (*p* < 0.05) were associated with higher risks of breast cancer after adjusting for body mass index and age (Figure 4). Conversely, lactate (OR: 0.689 (95% CI: 0.925-0.514)) (*p* < 0.05) and hydroxybutirate (OR: 0.696 (95% CI: 0.984-0.492)) (*p* < 0.05) were inversely associated with breast cancer risks after adjusting for body mass index and age (Figure 4).

**Figure 5:**
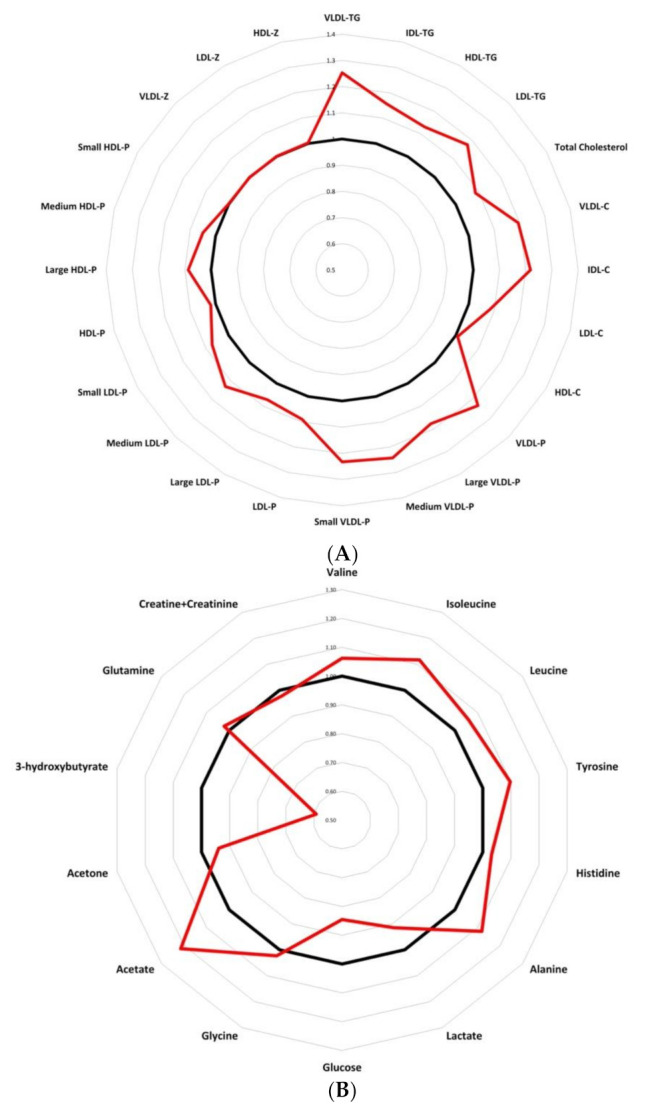
Radar charts presenting fold changes between control women and breast cancer patients, comparing quantitative variables: (**A**) lipidomic analysis showing an altered results for breast cancer patients compare to control women; (**B**) LMWMs showing a different spider chart for breast cancer patients compare to control women. Abbreviations: VLDL-c, very low-density lipoprotein cholesterol; IDL-c, intermediate-density lipoprotein cholesterol; LDL-c, low-density lipoprotein cholesterol; HDL-c, high-density lipoprotein cholesterol; Total-TG, total triglycerides; VLDL-TG, very low-density lipoprotein triglycerides; IDL-TG, intermediate-density lipoprotein triglycerides; LDL-TG, low-density lipoprotein triglycerides; HDL-TG, high-density lipoprotein triglycerides; VLDL-P, very low-density lipoprotein particle; large VLDL-P, large very low-density lipoprotein particle; medium VLDL-P, medium very low-density lipoprotein particle; small VLDL-P, small very low-density lipoprotein particle; LDL-P, low-density lipoprotein particle; large LDL-P, large low-density lipoprotein particle; medium LDL-P, medium low-density lipoprotein particle; Small LDL-P, Small low-density lipoprotein-particle; HDL-P, high-density lipoprotein-particle; Large HDL-P, large high-density lipoprotein particle; medium HDL-P, medium high-density lipoprotein particle; small HDL-P, small high-density lipoprotein particle; VLDL-z, very low-density lipoprotein size; LDL-z, low-density lipoprotein size, HDL-z, high-density lipoprotein size.

Finally, presenting a metabolic chart that allows us to easily classify breast cancer patients from control women, Figure 5 compares quantitative lipid particles (Figure 5A) and LMWMs (Figure 5B) between control woman and breast cancer patients. Data were analysed as fold changes. The graphic representation reveals a modified trend in triglyceride-rich lipoproteins and LMWMs in breast cancer patients. The results show an altered spider chart in breast cancer patients compared to control women, giving an interesting tool for pathologic breast cancer metabolism characterization.

## 4. Discussion

In this study, we explored the use of advanced NMR profiling to identify relevant metabolic alterations in breast cancer patient serum samples. Advanced NMR profiling showed that lipid metabolites were modified in breast cancer patients when compared to control healthy women. Specifically, total cholesterol and some of the transport proteins, such as IDL and LDL, were increased in breast cancer patients compared to control women. Interestingly, the numbers of lipoprotein particles were also modified in the serum of breast cancer patients. In addition, these lipoproteins also showed increased amounts of TGs (i.e., LDL-TG and HDL-TG).

The present findings are not only interesting but also important because there have been many controversies related to the roles of lipoproteins in cancer development [23,24,25]. In these studies, the authors analysed the role of cholesterol lipoprotein, but not the number or composition of the lipoproteins. In particular, our results showed that high levels of not only LDL cholesterol particles but also of triglyceride-enriched LDL and HDL molecules are associated with an increased risk of breast cancer. Consistent with our findings, Girona et al. 2019 previously proposed that HDL-TG should be considered as a biomarker for metabolic and cardiovascular risk, as well as a marker of HDL dysfunction. In the present study, we also observed altered HDL-TG compositions in breast cancer patients. In concordance, LDL has been shown to be an important molecule for promoting breast cancer cell migration and proliferation. On the contrary, HDL has also been described as an antioxidative molecule having antiproliferative effects in prostate cancer cells. Specifically, synthetic HDL has been shown to be able to mediate its antiproliferative function through apoA-I and phosphatidylcholine [26]. The antioxidant activity of HDL has also been demonstrated to be able to limit cell proliferation induced by reactive oxygen species (ROS), and this ability is lost in TG-enriched HDL. Moreover, the apoA-I levels have also been reported to be inversely correlated with TG composition in HDL [27]. Structurally, HDLs are spherical molecules when they consist of a cholesteryl ester and a triglyceride rich core but are discoidal when they contain mainly apoA-I **(20)**. These alterations in HDL composition and structure could lead to errors in the quantification of HDL-C, which could explain some of the discrepancies related to HDL-C and breast cancer.

TG-enriched particles have been related to altered metabolism and inflammation [27]. In our population, breast cancer patients showed an altered lipid metabolism and increased levels of TG-enriched particles, which could also result in an inflamed phenotype. Nevertheless, our findings revealed that LDL and HDL particles enriched in TG may increase the risk of breast cancer. As a result, altered compositions and amounts of LDL and HDL can potentially directly contribute to breast cancer risk. Collectively, these previous findings and the present results would suggest proatherogenic profiling of patients for breast cancer diagnosis and prognosis.

In addition, we also analysed some of the low molecular weight metabolites in order to define the influence of these molecules on breast cancer risk. Amino acids are used to supply nutrients in cancer cells to sustain their high proliferative rates [28]. We found that branched amino acids (valine *p* = 0.07; leucine *p* = 0.05; isoleucine *p* < 0.05) are associated with increased breast cancer risk. In fact, branched amino acids have been described as essential nutrients for cancer growth and function and as sources of energy for cancer cells [29]. As a consequence, branched amino acids could also be used as metabolic markers for breast cancer. In agreement, we also observed an imbalance in other LMWMs, such as tyrosine and alanine, in the sera of BC patients compared with control women. Tyrosine could be used by BC cells as building blocks for proteins, as well as an alternative cellular energy source. As tyrosine metabolism is important for cancer progression, and according to our results, the tyrosine level could also be classified as a breast cancer risk factor [30]. The role of alanine in cancer is less well understood, but as an amino acid it could play a part in cancer cell proliferation and survival. Nevertheless, our data clearly demonstrate that alanine is increased in breast cancer patients and can be a potential breast cancer risk factor [31]. On the contrary, lactate and hydroxybutyrate are inversely correlated with breast cancer risk. Lactate is inversely correlated with cancer risk, probably due to the fact that lactate can fuel tumour progression [32,33]. Moreover, hydroxybutyrate can potentially contribute to antiageing phenotypes, but its role in cancer remains controversial [34]. Several LMWMs were altered in the serum of BC patients and they can be used in conjunction with other lipid metabolites as reliable diagnostic and prognostic serum biomarkers for breast cancer.

This study provides clear correlations between triglyceride-enriched particles and breast cancer, although it has some limitations because the effects of hormone levels, post-menopausal state, and oral contraceptive use were not included due to the small size of this population. Further studies should be performed to increase the number of stratified breast cancer patients and to analyse the particles in these subpopulations.

## 5. Conclusions

In conclusion, our study has validated advanced NMR profiling as a valuable tool for detailed characterization of lipid and low molecular weight metabolites in cancer patient serum samples, and at the same time has contributed to a better understanding of the metabolic background of breast cancer. The use of advanced NMR profiling has allowed us to identify the relevant and specifically altered lipid and amino acid metabolites in breast cancer serum samples and to uncover specific metabolic signatures for early and reliable breast cancer patient diagnosis and prognosis.

## Figures and Tables

**Table 1:** Characteristics of the study group.

Study Group Data	Control (*n* = 69)	Breast Cancer (*n* = 171)	*p* Value
Clinical data			
Age	43 (38–54)	44 (37–50)	n.s
Children	2 (1–2)	2 (1–2)	n.s
Age of Menarche	12 (11–13)	12 (12–14)	n.s
Menopause (Yes, %)	30.3	20.2	n.s
BMI (Kg/m^2^)	24.02 (22.20–28.13)	25,07 (22.72–28.36)	n.s
Biochemical data			
Cholesterol (mg/dl)	194.18 ± 28.31	208.06 ± 31.40	<0.005
Apo A1 (mg/dl)	147.22 ± 28.66	147.64 ± 27.34	n.s
Apo B100 (mg/dl)	91 (78–104)	101 (85–118)	<0.05
Triglycerides (mg/dl)	76.55 (56.83–98.74)	86.73 (67.69–120.16)	<0.005
FABP4 (ng/mL)	13.085 (8.73–18.05)	17.53 (13.15–23.33)	<0.001
FABP5 (ng/mL)	6.12 (5.44–7.91)	7.00 (5.26–9.04)	n.s
CETP activity (pmol/µL)	47.87 (26.95–69.54)	51.98 (31.71–76.43)	n.s
CETP protein (ng/µL)	596.51 ± 163.39	579.63 ± 155.76	n.s
CD36 (pg/mL)	908.67 (762.00–1037.56)	897.00 (763.37–997.56)	n.s

Data are expressed as medians (IQR) for non-normally distributed data, means ± SD for normally distributed data, or percentages for categorical variables. The statistical tests used were Student’s t test (for data that were normally distributed), Mann–Whitney U test (for data that were not normally distributed), or chi-square tests (for data gathered as categorical variables). Abbreviations: ApoAI, apolipoprotein AI; ApoB100, apolipoprotein B100; BMI, body mass index; CETP, cholesteryl ester transfer protein; CD36, cluster of differentiation 36; IQR, interquartile range; SD, standard deviation. Baseline measurements for the study group. Data are expressed as means ± SD for normally distributed data, medians (IQR) for non-normally distributed data, and percentages for categorical variables. Student’s t test was used for data that were normally distributed, Mann–Whitney U test was performed for data that were not normally distributed, and chi-square tests were used for categorical variables. Abbreviations: ApoAI, apolipoprotein AI; ApoB100, apolipoprotein B100; BMI, body mass index; FABP4, fatty acid binging protein 4; FABP5, fatty acid binging protein 5; CETP, cholesteryl ester transfer protein; CD36, cluster of differentiation 36; IQR, interquartile range; SD, standard deviation.

## Data Availability

The data presented in this study are available on request from the corresponding author.

## References

[1] Siegel R.L., Miller K.D., Jemal A. (2018). Cancer statistics, 2018. CA Cancer J. Clin..

[2] Hanahan D., Weinberg R.A. (2011). Hallmarks of Cancer: The Next Generation. Cell.

[3] Beloribi-Djefaflia S., Vasseur S., Guillaumond F. (2016). Lipid metabolic reprogramming in cancer cells. Oncogenesis.

[4] Sun L., Suo C., Li S.-T., Zhang H., Gao P. (2018). Metabolic reprogramming for cancer cells and their microenvironment: Beyond the Warburg Effect. Biochim. Biophys. Acta. Rev. Cancer.

[5] Baenke F., Peck B., Miess H., Schulze A. (2013). Hooked on fat: The role of lipid synthesis in cancer metabolism and tumour development. Dis. Model. Mech..

[6] Cheng C., Geng F., Cheng X., Guo D. (2018). Lipid metabolism reprogramming and its potential targets in cancer. Cancer Commun..

[7] Liu Q., Luo Q., Halim A., Song G. (2017). Targeting lipid metabolism of cancer cells: A promising therapeutic strategy for cancer. Cancer Lett..

[8] Chimento A., Casaburi I., Avena P., Trotta F., De Luca A., Rago V., Pezzi V., Sirianni R. (2019). Cholesterol and Its Metabolites in Tumor Growth: Therapeutic Potential of Statins in Cancer Treatment. Front. Endocrinol..

[9] Simons K. (2000). How Cells Handle Cholesterol. Science.

[10] Ding X., Zhang W., Li S., Yang H. (2019). The role of cholesterol metabolism in cancer. Am. J. Cancer. Res..

[11] Orho-Melander M., Hindy G., Borgquist S., Schulz C.-A., Manjer J., Melander O., Stocks T. (2018). Blood lipid genetic scores, the HMGCR gene and cancer risk: A Mendelian randomization study. Int. J. Epidemiol..

[12] Abdelsalam K.E.A., Hassan I.K., Sadig I.A. (2012). The role of developing breast cancer in alteration of serum lipid profile. J. Res. Med. Sci..

[13] Hasija K., Bagga H.K. (2005). Alterations of serum cholesterol and serum lipoprotein in breast cancer of women. Indian. J. Clin. Biochem..

[14] Ashrafian H., Sounderajah V., Glen R., Ebbels T., Blaise B.J., Kalra D., Kultima K., Spjuth O., Tenori L., Salek R. (2020). Metabolomics—the stethoscope for the 21st century. Med. Princ. Pr..

[15] Holmes M.V., Millwood I.Y., Kartsonaki C., Hill M.R., Bennett D.A., Boxall R., Guo Y., Xu X., Bian Z., Hu R. (2018). Lipids, Lipoproteins, and Metabolites and Risk of Myocardial Infarction and Stroke. J. Am. Coll. Cardiol..

[16] Guaita-Esteruelas S., Saavedra-García P., Bosquet A., Borràs J., Girona J., Amiliano K., Rodríguez-Balada M., Heras M., Masana L., Gumà J. (2017). Adipose-Derived Fatty Acid-Binding Proteins Plasma Concentrations Are Increased in Breast Cancer Patients. Oncologist.

[17] Mallol R., Amigó N., Rodríguez-Gómez M., Ángel H.M., Vinaixa M., Plana N., Rock E., Ribalta J., Yanes O., Masana L. (2015). Liposcale: A novel advanced lipoprotein test based on 2D diffusion-ordered 1H NMR spectroscopy. J. Lipid. Res..

[18] Jeyarajah E.J., Cromwell W., Otvos J.D. (2006). Lipoprotein Particle Analysis by Nuclear Magnetic Resonance Spectroscopy. Clin. Lab. Med..

[19] Vinaixa M., Rodríguez M.A., Rull A., Beltrán R., Bladé C., Brezmes J., Cañellas N., Joven J., Correig X. (2010). Metabolomic Assessment of the Effect of Dietary Cholesterol in the Progressive Development of Fatty Liver Disease. J. Proteome. Res..

[20] Ouimet M., Barrett T.J., Fisher E.A. (2019). HDL and Reverse Cholesterol Transport. Circ. Res..

[21] Masana L., Girona J., Ibarretxe D., Rodríguez-Calvo R., Rosales R., Vallvé J.-C., Rodríguez-Borjabad C., Guardiola M., Rodríguez M., Guaita-Esteruelas S. (2018). Clinical and pathophysiological evidence supporting the safety of extremely low LDL levels—The zero-LDL hypothesis. J. Clin. Lipidol..

[22] Cedó L., Reddy S.T., Mato E., Blanco-Vaca F., Escolà-Gil J.C. (2019). HDL and LDL: Potential New Players in Breast Cancer Development. J. Clin. Med..

[23] Johnson K.E., Siewert K.M., Klarin D., Damrauer S.M., Chang K.-M., Tsao P.S., Assimes T.L., Maxwell K.N., Voight B.F., Program T.V.M.V. (2020). The relationship between circulating lipids and breast cancer risk: A Mendelian randomization study. PLoS Med..

[24] Guan X., Liu Z., Zhao Z., Zhang X., Tao S., Yuan B., Zhang J., Wang D., Liu Q., Ding Y. (2019). Emerging roles of low-density lipoprotein in the development and treatment of breast cancer. Lipids. Health Dis..

[25] Ganjali S., Ricciuti B., Pirro M., Butler A.E., Atkin S.L., Banach M., Sahebkar A. (2019). High-Density Lipoprotein Components and Functionality in Cancer: State-of-the-Art. Trends. Endocrinol. Metab..

[26] Ruscica M., Macchi C., Pavanello C., Corsini A., Sahebkar A., Sirtori C. (2018). Appropriateness of statin prescription in the elderly. Eur. J. Intern. Med..

[27] Girona J., Amigó N., Ibarretxe D., Plana N., Rodríguez-Borjabad C., Heras M., Ferré R., Gil M., Correig X., Masana L. (2019). HDL Triglycerides: A New Marker of Metabolic and Cardiovascular Risk. Int. J. Mol. Sci..

[28] Vettore L., Westbrook R., Tennant D.A. (2020). New aspects of amino acid metabolism in cancer. Br. J. Cancer..

[29] Ananieva E.A., Wilkinson A.C. (2018). Branched-chain amino acid metabolism in cancer. Curr. Opin. Clin. Nutr. Metab. Care.

[30] Nguyen T.N., Nguyen H.Q., Le D.-H. (2020). Unveiling prognostics biomarkers of tyrosine metabolism reprogramming in liver cancer by cross-platform gene expression analyses. PLoS ONE.

[31] Choi B.-H., Coloff J.L. (2019). The Diverse Functions of Non-Essential Amino Acids in Cancer. Cancers.

[32] Hirschhaeuser F., Sattler U.G., Mueller-Klieser W. (2011). Lactate: A Metabolic Key Player in Cancer. Cancer Res..

[33] de la Cruz-López K.G., Castro-Muñoz L.J., Reyes-Hernández D.O., García-Carrancá A., Manzo-Merino J. (2019). Lactate in the Regulation of Tumor Microenvironment and Therapeutic Approaches. Front. Oncol..

[34] Han Y.-M., Ramprasath T., Zou M.-H. (2020). β-hydroxybutyrate and its metabolic effects on age-associated pathology. Exp. Mol. Med..

